# Supplementary material for: Study protocol for a mixed methods convergent investigation of family domestic and sexual violence in multiple sclerosis and broader neurology in Australia
Source: PLoS One. 2026 Mar 13;21(3):e0344667. doi: 10.1371/journal.pone.0344667 (PMC12987488; doi:10.1371/journal.pone.0344667)
Supplement: S1 Table — (DOCX) [file pone.0344667.s001.docx]

**Supplementary Table 1**

| Risk | Outcome | Mitigation |
| --- | --- | --- |
| Exclusion and reduced access | Underreporting; discrimination; exclusion of those requiring support | Efforts will be made to optimise participation, including offering closed captioning, onsite support, and flexible participation modes. Participants unable to participate independently will be excluded, including those requiring interpreters. Interpreter use is considered a risk in FDSV research due to lack of specialised training, potential ties to community or culture, and the impact on participant comfort. All exclusions will be recorded and reported to inform future research strategies and equity considerations. |
| Participant distress or re-traumatisation during screening (Arm 1) | Emotional distress; disengagement from care | - Screening conducted by trusted clinicians trained in sensitive inquiry - Co-developed screening script with Family Violence experts and people with MS/CIS including victim survivors to ensure all language is sensitive and appropriate. - Immediate referral options, including MSNI psychologist support - Exclusion of individuals at risk of violence escalation (using MARAM evidence based risk indicators) - Technology safety assessed before participation  - In-person participation offered when remote participation is unsafe - Participants advised they may decline or defer questions or participation at any time |
| Privacy breach (Arms 1–4) | Confidential data accessed or misused | - All identifying data removed and replaced with study codes before storage and analysis - Secure data storage in compliance with local governance policies  - Mandatory reporting requirements will be explained at the beginning of every study arm. |
| Perpetrator becomes aware of victim-survivor’s participation | Worsening of violence | - Screening excludes participants with known risk of violence escalation (following MARAM evidence based risk indicators) - Screening integrated into routine clinical care and must be with the participant alone and in a private setting.  - Privacy for research is standard procedure in the clinic who routinely do research in sensitive areas including sexual health, reproduction history and terminations.  - Participants will be offered a script to explain the research was related to sexual/reproductive health if they are concerned about perpetrators wanting to know about the topic  - Screening and participation will be deferred to next appointment or not occur if a participant declines to be alone. Non-participation rates will be reported accordingly  - The study will not be advertised  - No written or electronic materials distributed including for final results given the risk of FDSV is dynamic. This includes verbal opt in consent models instead of written PICF documents where appropriate. - Safety is reassessed before every participation point given risk is dynamic.  - High-risk individuals referred to specialist services in Victoria following local protocols.  - Participants may opt out of data inclusion at any time |
| Underreporting | Misrepresentation of prevalence and response factors | - Screening script co-developed with experts and victim-survivors to ensure inclusive practice for people with neurological conditions and a trauma informed approach that supports victim survivors. - Screening conducted by long-term treating clinicians (acknowledging clinicians are best placed to follow up on disclosures of violence, and that disclosure is more likely to occur in a longitudinal trusting relationship). |
| Participant distress or re-traumatisation during survey (Arm 3) | Emotional distress or withdrawal | - Private survey completion with support options (in clinic or telehealth) - Dedicated distress protocols, including study pause or discontinuation - Referral options to MSNI psychology/psychiatry and external specialist supports - All participants provided with Victorian support contact options |
| Participant distress during interviews (Arm 4) | Re-traumatisation or emotional strain | - Interviews designed to be trauma-informed: breaks, question-skipping, and stopping supported - High-risk participants excluded via prior screening - Access to internal and external mental health supports during and post-interview - Dedicated distress and debriefing protocols have been made and will be followed accordingly (available on request) |
| Power imbalance – patients | Inhibition or discomfort disclosing to known clinicians | - Screening will be conducted by long-term treating clinicians, acknowledging that clinicians are best placed to respond to disclosures of violence and that disclosure is more likely to occur within a longitudinal and trusting therapeutic relationship. The wording and structure of the screening script, including language to reduce power imbalances, were developed in collaboration with HREC committees.  - Screening by unfamiliar researchers increases underreporting risk and limits access to ongoing support - Consent for Arms 3–4 is conducted by researchers with no therapeutic relationship to participants |
| Vicarious trauma or emotional stress – HCPs (Arm 5) | Burnout, emotional fatigue | - Interviews with flexible participation - Encouragement to access internal support (EAP, supervisors) - Dedicated distress protocols and debriefing |
| Power imbalance – HCPs | Edited responses or fear of judgement | - Only consultant-level neurologists included (not trainees) - Pre-interview scripts clarify the study is exploratory with no ‘correct’ responses and that the interview is not a knowledge test. - Participants may request a non-clinician interviewer - Identifying information removed prior to analysis and participants reassured about confidentiality |
| Vicarious trauma – researchers | Burnout or distress | - Research team supported via regular debriefs and supervision - Senior clinician mentorship and embedded psychology support - Access to university and hospital support services |
| Cognitive impairment and participation safety | Impaired capacity; inappropriate inclusion; safety concerns | Some individuals with MS or other neurological conditions may experience cognitive difficulties that affect decision-making capacity or safe participation. This may pose ethical and safety risks if not adequately assessed. - Capacity to consent and participate will be assessed by neurologists and neuropsychologists familiar with the participant - These assessments are part of routine care and will be incorporated into screening and enrolment processes - The research team includes clinical experts in neuroimmunology and cognitive impairment who are highly experienced in assessing and supporting safe, appropriate participation |
