# Supplementary material for: Study protocol for a mixed methods convergent investigation of family domestic and sexual violence in multiple sclerosis and broader neurology in Australia
Source: PLoS One. 2026 Mar 13;21(3):e0344667. doi: 10.1371/journal.pone.0344667 (PMC12987488; doi:10.1371/journal.pone.0344667)
Supplement: S3 File — (PDF) [file pone.0344667.s003.pdf]

55 Commercial Road
Melbourne VIC 3004
ABN 27 318 956 319

T +61 3 9076 2000
F +61 3 9076 2222
alfredhealth.org.au/research/
ethics-research-governance

Ethics & Research Governance Office
T +61 3 9076 3619
E research@alfred.org.au

AlfredHealth

ALFRED HOSPITAL ETHICS COMMITTEE
CERTIFICATE OF APPROVAL

This is to certify that

Project Number: 115129 (Local Reference: Project 207/25)

Project Title: From Risk to Response A Mixed-Methods Exploration of Violence Against People with MS and Health System Responses in Australian Clinics, with Broader Neurological Insights

Coordinating Principal Investigator: A/Professor Vilija Jokubaitis

was considered under the National Mutual Acceptance (NMA) scheme by the Alfred Hospital Ethics Committee on ***1-May-2025****, meets the requirements of the* National Statement on Ethical Conduct in Human Research 2023 *and was* ***APPROVED*** *on* ***17-Jun-2025***.

It is the Coordinating Principal Investigator's responsibility to ensure that all researchers associated with this project are aware of the conditions of approval and which documents have been approved.

The Coordinating Principal Investigator is required to notify the Alfred Hospital Ethics Committee, via amendment or progress report, of

- Any change to the project and the reason for that change, including an indication of ethical implications (if any);
- Any reportable safety issues according to the NHMRC's *Safety Monitoring and Reporting in Clinical Trials Involving Therapeutic Goods* (2016) or Alfred HREC requirements;
- Any reportable serious breaches according to the NHMRC's Reporting of serious breaches of Good Clinical Practice (GCP) or the protocol for trials involving therapeutic goods (2018);
- Any other unforeseen events or unexpected developments that merit notification;
- The inability of the Coordinating Principal Investigator to continue in that role, or any other change in research personnel involved in the project;
- A delay of more than 12 months in the commencement of the project; and
- Termination or closure of the project.

The conditions of Ethics Approval of this project are:

- All research projects approved by the Alfred Hospital Ethics Committee are subject to, and must be carried out in compliance with, the most recent applicable COVID-19 government and relevant institution's restrictions.
- Continuous insurance coverage must be maintained by the Sponsor, irrespective of the Sponsor type, throughout the project until the Final Report has been submitted to the Ethics Committee for acknowledgement and the project is ready to be archived. Therefore, if an insurance policy expires it is expected that it will be renewed under the same terms and submitted to the Ethics Committee.
- Submission each year of a Progress Report on the anniversary of Ethics Approval and on completion of the project.
- The HREC notes that it is the responsibility of the researchers to ensure that they and any other people or entities involved in the conduct of the study comply with all applicable laws regarding the conduct of the study, including
    - privacy legislation, both Commonwealth and state/territory.

The Ethics Committee may conduct an audit at any time.

All research subject to the Alfred Hospital Ethics Committee review must be conducted in accordance with the *National Statement on Ethical Conduct in Human Research 2023*, as well as the Alfred Hospital Ethics Committee's requirements.

The Alfred Hospital Ethics Committee is a properly constituted Human Research Ethics Committee in accordance with the *National Statement on Ethical Conduct in Human Research 2023.*

SPECIAL CONDITIONS of ETHICS APPROVAL

1. **The Alfred Hospital Ethics Committee has granted a waiver of consent, in accordance with the criteria in Section 2.3.10 (a) to (i) of the National Statement 2023.**

2. **The Alfred Hospital Ethics Committee has approved the opt-out approach in this research project, noting compliance with Section 2.3.6 of the National Statement 2023**

APPROVED DOCUMENTS
Documents reviewed and approved:

Document	*Version*	*Date*
Protocol	1.1	23-May-2025
Appendix 1 – People with MS (and Epilepsy/Headache)	-	-
Appendix 2 – HCPs	-	-
Appendix 3 – Surveys + Forms	-	-
Appendix 4 – Distress protocol for research team	-	-
Appendix 6 – Electronic Consent	-	-
Participant Information Sheet & Consent Form – Patient	1	5-May-2025
Participant Information Sheet & Consent Form – HCP	1	5-May-2025
1.4 Data collection for people with Epilepsy or Headache	-	-
2.2 Interview Guide for HCPs	-	-
2.2b Additional Data Survey for HCPs	-	-

APPROVED SITES
Approval is given for this research project to be conducted at the following sites:

- Monash University (Site PI: A/Professor Vilija Jokubaitis)
- The Alfred – Alfred Health (Site PI: A/Professor Anneke van der Walt)

The Alfred Hospital Ethics Committee has approved the study but does not take responsibility for research governance processes at the participating sites. It is the responsibility of each participating site to create and implement research governance practices to adequately authorise, monitor and oversee the conduct of the study at their site.

Site-Specific Assessment (SSA) Authorisation
This Ethics Approval Certificate constitutes ethical approval only. This project cannot commence at any site, including Alfred Health, until separate site authorisation (research governance authorisation) has been obtained.

The HREC wishes you and your colleagues every success in your research.

SIGNED:

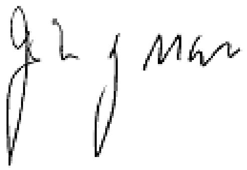

Professor John McNeil
Chair, Ethics Committee

Please quote project number and title in all correspondence.
