# Supplementary material for: Study protocol for a mixed methods convergent investigation of family domestic and sexual violence in multiple sclerosis and broader neurology in Australia
Source: PLoS One. 2026 Mar 13;21(3):e0344667. doi: 10.1371/journal.pone.0344667 (PMC12987488; doi:10.1371/journal.pone.0344667)
Supplement: S4 File — (PDF) [file pone.0344667.s004.pdf]

Monash University Human Research Ethics Committee

Confirmation of Registration

**Project Number:**	48580
**Project Title:**	From Risk to Response A Mixed-Methods Exploration of Violence Against People with MS and Health System Responses in Australian Clinics, with Broader Neurological Insights
**Chief Investigator:**	Assoc Professor Vilija Jokubaitis
**Registration Date:**	23/07/2025

Terms:

1. Registration is valid whilst you hold a position at Monash University, and approval at the Other HREC is current.
2. This notification does not constitute HREC approval. It is the responsibility of the Chief Investigator to ensure that approval from the Other HREC continues for the duration of the research.
3. Retention and storage of data: The Chief Investigator is responsible for the storage and retention of the original data pertaining to this project in accordance with the *Australian Code for the Responsible Conduct of Research*.

Kind Regards

Professor William Sievert

Chair, MUHREC

CC: Ms Cassie Nesbitt

List of registered documents:

Document Type	File Name	Date	Version
Supporting Documentation	Protocol 207_25 from_risk_to_response_FDSV in MS_JULY 2025	23/07/2025	1
Supporting Documentation	Protocol 207_25 from_risk_to_response_FDSV in MS_MAY_23_2025	23/07/2025	2
Supporting Documentation	SSA 207 25	23/07/2025	1
Supporting Documentation	Alfred ERA revisions and feedback FINAL	23/07/2025	1
Primary HREC Certificate of Approval	115129 (Local Reference 207-25) Ethics Approval Certificate 17-Jun-2025	23/07/2025	1
Primary HREC Application	project 207_25_NEAF	23/07/2025	1
Explanatory Statement	PICF_FDSVinMS_HCP_AlfredHealth_clean may 2025	23/07/2025	1
Consent Form	PICF-FDSVinMS_pw_ms epi headache_AlfredHealth_clean_may2025	23/07/2025	1
