# Supplementary material for: Study protocol for a mixed methods convergent investigation of family domestic and sexual violence in multiple sclerosis and broader neurology in Australia
Source: PLoS One. 2026 Mar 13;21(3):e0344667. doi: 10.1371/journal.pone.0344667 (PMC12987488; doi:10.1371/journal.pone.0344667)
Supplement: Attachment — Submitted filename: Response to reviewers.docx [file pone.0344667.s005.docx]

Dear Dr Yordanis Enríquez Canto

PLOS One Academic Editor,

Re: PONE-D-25-65254
Study protocol for a mixed methods convergent investigation of family domestic and sexual violence in multiple sclerosis and broader neurology in Australia

Thank you for the opportunity to submit a revised version of our manuscript. We are grateful to you and the reviewers for the careful assessment of our protocol. We appreciate the reviewers’ recognition of the importance of the topic, the comprehensive literature review, the robustness of the mixed methods design, and the meaningful involvement of victim-survivors in co-development.

We have revised the manuscript to address all points raised. Key revisions include clarification of the clinician led screening and consent processes and safeguards to minimise perceived influence, specification of additional demographic and clinical variables and how they will be incorporated across study arms, expanded feasibility and safety process indicators in the quantitative analysis plan, strengthened justification of feasibility based quantitative sample size targets, clearer definition and operationalisation of thematic saturation with an audit trail approach, inclusion of an explicit missing data approach, and an expanded Background and Research Context section summarising the international MS literature on IPV and FDSV.

All changes have been tracked.

A detailed, point by point response is provided in the table below, with corresponding manuscript locations.

Sincerely,

Dr Cassie Nesbitt
on behalf of all authors

|  | Reviewer feedback | Response | Ref |
| --- | --- | --- | --- |
| - | The reviewers commend the importance of your study topic, the comprehensive literature review, and the robust mixed-methods design, as well as the meaningful involvement of victim-survivors in co-development. They agree that your study aims are relevant and achievable and that the methodology is appropriate for addressing this important issue. | We thank the reviewers for their encouraging feedback.  We particularly appreciate your acknowledgement of the victim-survivors with MS on the research team, whose lived expertise has directly informed the co-design and conduct of the study. Their courage and determination to use lived experience to drive positive change for others merits explicit recognition, and we are grateful you took the time to highlight this. | - |
| 1 | Clarify the informed consent process, particularly addressing any concerns about potential influence when screening and consent are obtained by treating clinicians, given the sensitive nature of family violence. | We have expanded the Consent section to clarify clinician led screening and verbal opt in consent for Arms 1 and 2 and to address perceived influence. Clinicians use a standardised script stating participation is voluntary and will not affect care, and participants may skip questions or pause, stop, or defer at any time. Where preferred, or where clinically more appropriate, consent may be completed with a trained non treating team member. | Consent pages 15-16 |
| 2 | Specify whether data on sexual orientation, disease-modifying therapy use, and years of education will be collected, and if so, how these variables will be incorporated. | Sexual orientation and education level will be collected in Arm 3 as part of the survey demographics. Education will be recorded as highest level attained, which serves as a proxy for years of education in the Australian context.  These variables are not routinely captured in clinical records or MSBase and will therefore be incorporated in survey-based analyses in Arm 3, not in the linked Arm 2 dataset.  DMT use will be captured from MSBase and clinical records as part of routine MS history and will be incorporated in the linked Arm 2 analyses alongside other MS clinical variables. The original draft did not state DMT use explicitly and this omission has been corrected. | Study arms 2-3 pg 16-17 |
| 3 | Provide greater detail on how the feasibility of family domestic and sexual violence (FDSV) screening in neurology research will be evaluated within your data analysis plan, including aspects of safe disclosure, safety management, and support pathways. | We expanded the quantitative analysis plan to define feasibility using descriptive process and safety indicators, covering safe approach and uptake, safe disclosure setting, and the frequency and type of safety management and support pathway actions initiated following screening, with denominators reported for each indicator. | Feasibility, safe disclosure, and response pathways  Pg 17-18 |
| 4 | Elaborate on the calculation of the sample size in the quantitative arm of the study. | Quantitative sample size is now justified as feasibility based. The screening target of approximately 1,000 is derived from annual clinic throughput of about 1,000 individual people with MS over a 12-month recruitment window, noting some attend more than once, and constrained by the requirement to approach only when the person is private and alone.  The survey target of approximately 100 is stated as pragmatic expert consensus on likely uptake for a sensitive topic, and the protocol now specifies an anticipated range of 70 to 150 completed surveys.  The protocol also now defines thematic saturation as the point where additional interviews yield no new substantive codes that alter the coding framework or interpretation, and specifies iterative assessment within participant groups using an audit trail of dated codebook versions, analytic memos, and analysis meeting notes, with a minimum of approximately 30 interviews for depth and additional interviews only if new substantive concepts continue to emerge. | Sample size  Pg 11-12 |
| 5 | Describe how thematic saturation will be assessed in the qualitative arm, including details on how "thematic saturation" is defined and calculated.  Clarify the rationale for the target number of approximately 100 surveys and specify any estimated range (minimum and maximum) for survey numbers. |  |  |
| 6 | Outline your approach to managing missing or incomplete data, considering the potential for a substantial amount of such data in this extensive study. | We have added a Missing data statement to the quantitative analysis plan. Missingness will be reported by study component and variable with denominators for all estimates. Missingness for routinely captured MS variables is anticipated to be low based on established site recording practices, whereas survey item nonresponse is uncertain given the sensitive content and option to skip questions and will be interpreted as a feasibility and acceptability finding. No imputation will be undertaken, and sensitivity analyses will be conducted where appropriate. | Quant data analysis  Pg 18-19 |
| 7 | Expand the Background/Context section to include results from other studies worldwide on intimate partner violence (IPV) and family domestic and sexual violence (FDSV) among people with multiple sclerosis, to further support the research rationale. | We expanded the Background and Research Context sections to summarise the international MS literature on IPV and FDSV, including study settings, measurement approaches, and prevalence estimates, and added recent large multicentre data from Italy (2026) alongside existing evidence from the United States, Iran, Sweden, and Norway. | Research context pages 4-6 |
